# Exploring the Link between Leaky-Gut-Related Markers and Metabolic Health in a Large Dutch Adult Population

**DOI:** 10.3390/metabo11120877

**Published:** 2021-12-16

**Authors:** Hiroyuki Hoshiko, Edith J. M. Feskens, Els Oosterink, Renata M. C. Ariens, Jurriaan J. Mes, Nicole J. W. de Wit

**Affiliations:** 1 HE Center, Suntory MONOZUKURI Expert Ltd., Kyoto 619-0284, Japan; 2 Human Nutrition and Health, Department of Agrotechnology and Food Sciences, Wageningen University, 6708 WE Wageningen, The Netherlands; edith.feskens@wur.nl; 3 Wageningen Food and Biobased Research, Wageningen University & Research, 6708 WG Wageningen, The Netherlands; els.oosterink@wur.nl (E.O.); renata.ariens@wur.nl (R.M.C.A.); jurriaan.mes@wur.nl (J.J.M.); nicole.dewit@wur.nl (N.J.W.d.W.)

**Keywords:** leaky gut, metabolic health, leaky-gut markers

## Abstract

A leaky gut can trigger chronic inflammation and poses a primary risk for metabolic diseases. This study established a relationship between intestinal integrity (leaky gut) and metabolic health in a general population. Leaky-gut markers (LGMs) were studied in a large population of Dutch adults with a broad spectrum of metabolic health. This study enrolled 500 individuals selected within the NQplus cohort study (*n* = 2048) by stratified randomization, based on waist circumference, fasting glucose, and high-density lipoprotein (HDL) cholesterol to obtain a representative and balanced population in terms of metabolic health parameters, sex (male/female), and age (<54/≥54 years). LGMs—zonulin, lipopolysaccharide-binding protein (LBP), and soluble CD14 (sCD14)—were measured in EDTA plasma or serum. Zonulin was most strongly associated with metabolic health. Zonulin and LBP were most strongly associated with the inflammatory marker C-reactive protein (CRP). The quartile analysis for zonulin and LBP showed that most metabolic health parameters and CRP levels increased from Q1 to Q4, with significant differences between quartiles, except for markers related to glucose homeostasis (glucose and glycated hemoglobin A1c (HbA1c)). Associations between LGMs and metabolic health parameters in this large Dutch adult population indicate that LGMs are valuable markers for identifying people at risk of a leaky gut and subsequent chronic inflammation linked to metabolic disorders.

## 1. Introduction

Obesity, especially when characterized by a high waist circumference [1], is primarily linked to an unhealthy metabolic profile in humans [2]. Obesity is associated with chronic inflammation in the adipose tissue, which can lead to metabolic dysfunction [3]. In addition to obesity, a range of other factors may also contribute to chronic inflammation, such as a leaky gut [4]. Recent hypotheses have linked a leaky gut to chronic inflammation due to the translocation of microbiome-derived lipopolysaccharide (LPS) into the bloodstream, possibly leading to metabolic endotoxemia. This LPS translocation may trigger inflammatory activation, eliciting a chronic low-grade pro-inflammatory and pro-oxidative stress status associated with chronic metabolic diseases linked to metabolic syndrome [5]. LPS in the blood binds to a lipopolysaccharide-binding protein (LBP), passes to sCD14, and subsequently transfers to myeloid differentiation factor 2 (MD2) and toll-like receptor 4 (TLR4), which triggers inflammatory pathways [6]. Recent studies have shown that circulating levels of LBP and sCD14 are correlated with inflammatory markers in obese older subjects [7], and that they are elevated in metabolically unhealthy subjects compared to metabolically healthy ones [4]. These findings indicate that a leaky gut has a direct or indirect role in the development of metabolic disorders related to metabolic syndrome [8]. 

In our previous study, we measured a broad range of leaky-gut-related biomarkers and studied their association with metabolic health in a limited group of Dutch adults with distinct metabolically healthy and metabolically unhealthy profiles [4]. We also conducted a pilot observational study to explore whether metabolic health could be linked to paracellular intestinal permeability, as assessed by a multi-sugar permeability test, and whether paracellular intestinal permeability is correlated with LGMs [9]. At baseline, metabolic health parameters and LGMs showed significant correlations. The multi-sugar gut permeability markers did not correlate with LGMs (in either plasma or serum), leading to the hypothesis that transcellular translocation and/or lipoprotein-related transportation is a more likely mechanism underlying the association between LGMs and metabolic health [9]. In both studies, zonulin was the most relevant leaky-gut marker, and showed the most pronounced relation with metabolic health. Zonulin was reported to be associated with obesity-associated insulin resistance [10], and increased serum zonulin and LPS was previously found in patients with type 2 diabetes mellitus [11]. Nonetheless, the association between LGMs and metabolic health parameters in general populations remains largely unclear. In this study, we evaluated the application of zonulin, as it is associated with intestinal permeability, and LBP and sCD14 as biomarkers for endotoxin-related translocation in a larger cohort of the adult Dutch population with a broad spectrum of metabolic parameters. This study’s findings help strengthen the evidence of the relationship between intestinal integrity and metabolic health in general populations. Additionally, the level of CRP was measured to link the LGMs to an inflammatory status, as low-grade inflammation is often associated with metabolic syndrome.

## 2. Results

### 2.1. Characteristics of the Study Population

Five hundred individuals were selected within the NQplus cohort study by stratified randomization for waist circumference, fasting glucose, and HDL cholesterol to obtain a large adult population with a broad range in metabolic health. Table 1 shows the range of metabolic health profiles (the minimum and maximum values). The anonymized dataset is summarized in Table S1.

### 2.2. Measurement of LGMs

The leaky-gut markers LBP, sCD14, and zonulin were tested in serum and EDTA plasma samples for the selected 500 individuals. Table 2 shows the minimum, maximum, and mean values (absolute values) for each marker. There is a large difference between the lowest and highest values detected. The standard deviation (SD) values also indicated that substantial variation can be found between the individuals. As our previous study indicated that specifically sCD14 can show sex- and age-specific associations with metabolic health, we distinguished between men and women, as well as younger and older individuals, for the LGM measurements (Table 3 and Table 4). As a result, sCD14 showed significantly higher levels in women and older individuals (Mann–Whitney U test, *p* < 0.05). As expected, for LBP and zonulin, the mean levels are highly comparable between the sex and age groups.

### 2.3. Relationship between LGMs and Metabolic Health

**Figure 1:**
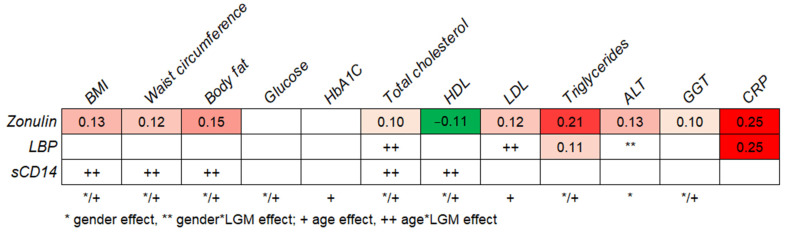
Stepwise linear regression analyses to test the relationship between leaky-gut markers (LGMs) and metabolic markers. BMI: body mass index; HbA1c: glycated hemoglobin A1c, HDL: high-density lipoprotein, LDL: low-density lipoprotein, ALT: alanine aminotransferase, GGT: gamma-glutamyl transpeptidase, CRP: C-reactive protein, LBP: lipopolysaccharide-binding protein, and sCD14: soluble CD14.

Stepwise linear regression analyses were performed to determine the relationship between LGMs and the metabolic health parameters. Age and sex were also included in the analyses. For most metabolic markers, we found a significant main effect for age and/or sex (Figure 1). As for LGMs, zonulin was significantly associated with most of the metabolic parameters, especially with triglycerides and the inflammatory marker CRP (Figure 1). LBP showed the strongest relation with CRP. For total cholesterol, LDL cholesterol, and ALT, effects of LBP were found to interact with sex or age. Therefore, follow-up linear regression analysis for these metabolic markers was performed separately for younger (<54 years) and older people (≥54 years) or men and women. LBP showed a relation with total cholesterol (β = −0.13, *p* = 0.03), but only in older individuals. For LDL cholesterol and ALT, no clear differences between the age and sex groups could be found. Moreover, no significant effects of sCD14 on metabolic parameters were found. For BMI, waist circumference, body fat, total cholesterol, and HDL cholesterol, the effects of sCD14 were found to interact with age. Separate linear regression analyses for younger (<54 years) and people (≥54 years) older showed that a positive relation between sCD14 and total cholesterol was only present in older individuals (β = 0.21, *p* = 0.001). In the same older population, an unexpected positive association was found with HDL cholesterol (β = 0.21, *p* = 0.001), and negative relationships with the BMI (β = −0.17, *p* = 0.008) and waist circumference (β = −0.21, *p* = 0.001) were found. We found a positive association between body fat and sCD14 (β = 0.25, *p* = 0.04), but only in the population below 40 years of age. 

The standardized coefficient ß of the final model is only reported if *p* < 0.05. LGMs were the independent variables, and the metabolic markers were the dependent variables. Red indicates a positive relationship (a more intense color means a stronger relationship), and green indicates a negative relationship.

### 2.4. Quartile Analyses

For quartile analysis, the total population of this study was ranked for a certain marker and divided into four equal groups. The results of quartile analyses for zonulin, LBP, and sCD14 are shown. For quartiles based on zonulin (Table 5) and LBP (Table 6), we found that most metabolic health parameters and the inflammatory marker CRP increased from Q1 to Q4 and showed significant differences between the quartiles, except for the markers related to glucose homeostasis (glucose and HbA1c). Quartile analysis for sCD14 (Table 7) revealed that fewer metabolic markers were significantly different compared to the zonulin and LBP quartiles, but total cholesterol showed an interesting profile, and CRP and HbA1c levels increased from Q1 to Q4. 

## 3. Discussion

In this study, zonulin, the most relevant biomarker selected from our previous study, and LBP and sCD14 were studied in a large population of Dutch adults representing a broad spectrum of metabolic health. Zonulin is associated with intestinal permeability [4], and LBP and sCD14 are biomarkers for endotoxin-related translocation. This larger population of the NQplus cohort study was chosen to investigate the relationship between intestinal integrity and an unhealthy metabolic profile related to metabolic syndrome for the general population (and not only for the most severely affected part of the population [4]). Additionally, CRP levels were measured to link the leaky-gut-related biomarkers to the inflammatory status, as low-grade inflammation is often associated with metabolic syndrome. The mean values of the metabolic data indicate that a decently healthy population was included in the NQplus cohort study along with this study. Only 22 men and 13 women could be diagnosed with metabolic syndrome based on the combination of waist, fasting glucose, and HDL cholesterol (central obesity (defined as waist circumference ≥94 cm for Europid men and ≥80 cm for Europid women, with ethnicity-specific values for the other groups), raised fasting plasma glucose ≥5.6 mmol/L, and reduced HDL cholesterol <1.03 mmol/L in men and <1.29 mmol/L in women) [12]. This inclusion of a healthy population in this study may have resulted from the chosen exclusion of people who were already diagnosed with diseases such as diabetes, cardiovascular disease, and liver dysfunction. 

Serum zonulin levels reflect gut permeability via the intestinal epithelial paracellular route [13]; however, we could not verify this in our observational study in which the results of a multi-sugar permeability test, reflecting paracellular permeability along the intestinal tract, were compared to blood LGM levels in connection to metabolic health [9]. No significant correlations were found between the two different methods of measuring gut permeability. Furthermore, in this previous observational study, we found minimal links between paracellular permeability, as measured with a multi-sugar permeability test, and metabolic health, whereas zonulin showed significant correlation with various metabolic health parameters. Although the association in this current study was not as strong as in our previous study in which only the most metabolically healthy and unhealthy individuals of the NQplus cohort were included [4], most associations for zonulin and metabolic health markers are comparable between all three studies. This is an important finding, as it shows that the association between zonulin and metabolic health is robust and applicable for a broad range of the Dutch adult population. In the stepwise regression analysis, zonulin was significantly associated with most of the metabolic parameters, especially with triglycerides and the inflammatory marker CRP. This implies that zonulin, and thus gut permeability, can indeed be linked to chronic inflammation and metabolic health. A potential link between zonulin and metabolic parameters is supported by a study by Bodil Ohlsson et al., who found that higher zonulin levels were associated with an increased risk of overweight, obesity, and hyperlipidemia [14]. Fasano et al. recently showed that zonulin is implicated in a variety of chronic inflammatory diseases [15]. For zonulin quartiles, most metabolic health parameters showed significant differences between the quartiles, except for markers related to glucose homeostasis (glucose and HbA1c). This fits the data from stepwise regression analysis as no significant associations were found between zonulin and glucose or HbA1c. A previous study, however, clearly revealed that circulating zonulin levels are associated with insulin sensitivity measured by an intravenous glucose tolerance test [10]. This may indicate that a glucose loading test is necessary to evaluate a potential link between this marker and glucose homeostasis.

LBP was suggested as a clinical marker of endotoxemia [16]. LBP showed a pronounced association with CRP. This finding suggests that LBP is linked to CRP, and thus chronic inflammation, and that this might indirectly link LBP to metabolic health. Quartile analysis for LBP also supported these data, as most metabolic parameters were found to be significantly different between the LBP quartiles. Previous studies also reported that LBP has a more pronounced correlation with inflammatory markers than with metabolic markers, and a substantial correlation was also found with triglycerides [17]. This could be indicative of the association between LPS and chylomicron-driven translocation/transportation of LPS. It has recently been reported that dietary fat can promote the absorption of LPS via the enhanced production of chylomicrons [18,19,20]. Furthermore, there are indications that saturated and polyunsaturated dietary fat differentially regulate intestinal LPS translocation, where a higher translocation was seen with saturated fat [21]. However, as the blood samples used in this study were from patients who fasted overnight, and almost all chylomicrons were cleared from the blood, finding stronger correlations and expanding our knowledge of the potential role of chylomicrons would require postprandial blood sampling, which was not implemented in the NQplus study.

sCD14 acts as a shuttle to transfer LPS to HDL to neutralize the toxicity of LPS [22]. The HDL, in combination with sCD14, could therefore have an additive/synergistic protective effect on the translocated LPS before it can induce inflammatory triggers. In this study, sCD14 showed a positive association with HDL and a negative association with BMI and waist circumference in the older population. This is also supported by the quartile analysis for sCD14, which showed increasing values of HDL and decreasing values of BMI from Q1 to Q4. A previous study showed that the circulating levels of sCD14 were significantly increased in obese subjects compared to healthy non-obese subjects [23]. In this study, we found a positive (non-significant) correlation between circulating levels of sCD14 and HDL cholesterol. BMI and waist circumference showed a positive correlation with sCD14 in previous studies, which is in contrast with our findings. The inclusion of a substantially younger population might partly explain this discrepancy, as in our study, significant associations were only detectable in the older population. In general, the effect of age on intestinal permeability is still debatable as different studies have reported conflicting results. Intestinal permeability may be more related to increasing co-morbidities than to increasing age.

This study confirms the correlation between LGMs, especially zonulin, with various metabolic markers in a large group of adults with a broad metabolic health spectrum. This thereby strengthens the evidence of the relationship between intestinal integrity and metabolic health in general populations.

## 4. Materials and Methods

### 4.1. Study Participants

**Figure 2:**
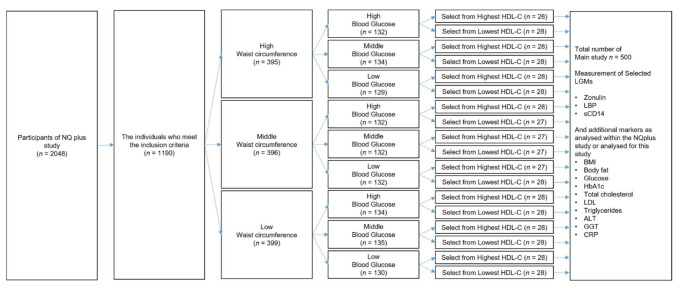
Schematic overview of the individuals selected from the NQplus study. Following this scheme, 500 individuals with a broad metabolic profile were selected from the NQplus study. LGM: leaky-gut marker, BMI: body mass index, HbA1c: glycated hemoglobin A1c, HDL: high-density lipoprotein, LDL: low-density lipoprotein, ALT: alanine aminotransferase, GGT: gamma-glutamyl transpeptidase, CRP: C-reactive protein, LBP: lipopolysaccharide-binding protein, and sCD14: soluble CD14.

The NQplus cohort study was previously conducted by Wageningen University’s Division of Human Nutrition [24]. The NQplus study was approved by the Medical Ethical Committee of Wageningen University (NL34775.081.10) and was conducted according to the guidelines laid down in the Declaration of Helsinki. All participants gave written informed consent. This cohort study included 2048 individuals from whom the data regarding demography, food intake, and metabolism were collected, in addition to plasma and serum samples, and stored for future analyses. Patients with a history of diabetes mellitus, myocardial infarction, heart failure, kidney dysfunction, liver dysfunction (cirrhosis, hepatitis), gastrointestinal disorders (stomach ulcer, ulcerative colitis, Crohn’s disease, and celiac disease), ethanol intake ≥60 g/day, and current smokers were excluded from the study. Additionally, the 40 most metabolically healthy and 40 most unhealthy individuals, who were already analyzed in our previous study, in whom we identified the most relevant LGMs, were excluded [4]. Subsequently, 500 individuals were selected by stratified randomization to obtain a representative and balanced population in terms of sex, age, and metabolic health parameters based on waist circumference, glucose, and HDL cholesterol, according to the scheme shown in Figure 2. Stored serum and EDTA plasma samples from these 500 selected individuals were used for further analyses in the present study.

### 4.2. Measurement of the Markers

LGMs were measured in the serum or EDTA plasma samples of the 500 individuals selected from the NQplus study [22]. These samples were collected in the fasting state (after an overnight fasting period) and were stored at −80 °C at the Division of Human Nutrition of Wageningen University. Enzyme-linked immunosorbent assays (ELISA) were used to measure zonulin (K5601, Immundiagnostik AG, Bensheim, Germany), LBP (HK315-02, Hycult Biotech, Uden, The Netherlands), and sCD14 (HK320-02, Hycult Biotech), according to the manufacturers’ protocol. After measurement of LGMs, CRP, a marker for inflammation, was measured in EDTA plasma (CRP range: 0.25–16 ng/mL) (EC1001-1, Assaypro LLC, St. Charles, MO, USA). All samples were analyzed in duplicates.

### 4.3. Statistical Analyses

The statistical analyses were conducted using SPSS software version 22 (IBM Corp., Armonk, NY, USA). Only the values with a coefficient of variation <20% in the ELISA were considered adequate and were included in the statistical analyses (for all markers). Outliers were also included (analyzed by boxplots (IQR)) as no reasonable arguments were available to exclude these samples from further analyses. We verified that these outliers did not substantially affect the statistical outcomes, as we performed analyses with and without them. The normal distribution of the markers was checked using the Shapiro–Wilk test. For stepwise regression analyses, values were log_10_-transformed for all markers, and “0” or “below detection limit” were adjusted to half of the lowest value that could be measured. The metabolic markers were then set as dependent variables and the LGMs as fixed predictors (independent variables). To also explore interactions with age and sex, centered values were first calculated for the log-transformed LGM values and sex and age. The interaction was then quantified using log-centered LGM × centered sex or centered age. These interaction values were then also included in the stepwise regression analyses as independent variables.

## 5. Conclusions

Altogether, our study’s findings indicate that LGMs, especially zonulin, are applicable as additional metabolic health markers for the general Dutch adult population. It also shows that zonulin can be used to identify people at risk of leaky gut and subsequent chronic inflammation linked to metabolic disorders.

## Figures and Tables

**Table 1:** General and metabolic characteristics of the individuals selected from the NQplus study (*n* = 500).

Metabolic Parameter	Minimum	Maximum	Mean (SD)
Age (years) *	20	77	52 (11.7)
BMI (kg/m^2^)	16.6	40.5	24.7 (3.4)
Waist circumference (cm)	64.0	124.0	89.4 (10.9)
Body fat (%)	5.1	54.0	29.2 (8.5)
Glucose (mmol/L)	3.9	8.9	5.4 (0.6)
HbA1c (mmol/mol)	22.0	49.7	35.7 (3.2)
Total cholesterol (mmol/L)	2.0	8.6	5.52 (1.1)
HDL (mmol/L)	0.7	3.7	1.7 (0.6)
LDL (mmol/L)	0.7	6.0	3.3 (0.9)
Triglycerides (mmol/L)	0.3	4.9	1.2 (0.7)
ALT (U/L)	9.2	209.0	26.8 (14.6)
GGT (U/L)	5.2	253.6	23.9 (23.0)
CRP (mg/mL)	0.001	129.9	2.57 (7.4)

* Sex equally divided amongst the group (female:male, *n* = 250:250). BMI: body mass index, ALT: alanine aminotransferase, GGT: gamma-glutamyl transpeptidase, HbA1c: glycated hemoglobin A1c, HDL: high-density lipoprotein, LDL: low-density lipoprotein, CRP: C-reactive protein.

**Table 2:** LGM levels in the individuals selected from the NQplus study (*n* = 500).

LGM	Minimum	Maximum	Mean (SD)
Zonulin (ng/mL)	14.7	227.9	32.0 (13.4)
LBP (µg/mL)	0.7	26.0	9.6 (3.5)
sCD14 (µg/mL)	0.7	2.7	1.4 (0.3)

LGM: leaky-gut marker, LBP: LPS-binding protein, sCD14: soluble CD14. For LBP: *n* = 0, sCD14: *n* = 1, and Zonulin: *n* = 1, samples were considered inadequate and were excluded from the statistical analyses.

**Table 3:** LGM levels in male (*n* = 250) and female (*n* = 250) individuals separately.

LGM	Male	Female	*p*-Value *
Zonulin (ng/mL)	32.4 (12.0)	31.6 (14.7)	0.12
LBP (µg/mL)	9.8 (3.2)	9.5 (3.8)	0.37
sCD14 (µg/mL)	1.37 (0.3)	1.52 (0.3)	<0.001

LGM: leaky-gut marker, LBP: LPS-binding protein, sCD14: soluble CD14. For LBP: *n* = 0, sCD14: *n* = 1, and Zonulin: *n* = 1, samples were considered inadequate and were excluded from the statistical analyses. Data in Table 3 are presented as mean (SD). * Significance by the Mann–Whitney U test.

**Table 4:** LGM levels in the younger (*n* = 247) and older (*n* = 253) individuals separately.

LGM	Younger (<54 y)	Older (≥54 y)	*p*-Value *
Zonulin (ng/mL)	31.0 (8.8)	33.0 (16.7)	0.84
LBP (µg/mL)	9.4 (3.6)	9.9 (3.4)	0.29
sCD14 (µg/mL)	1.39 (0.3)	1.50 (0.3)	0.02

LGM: leaky-gut marker, LBP: LPS-binding protein, sCD14: soluble CD14. For LBP: *n* = 0, sCD14: *n* = 1, and Zonulin: *n* = 1, samples were considered inadequate and were excluded from the statistical analyses. Data in Table 4 are presented as mean (SD). * Significance by the Mann–Whitney U test.

**Table 5:** The quartile analyses for zonulin including all individuals (*n* = 500).

Metabolic Parameter	Q1	Q2	Q3	Q4	*p*-Value *
BMI (kg/m^2^)	23.9 (2.9)	24.2 (3.0)	25.2 (3.4)	25.9 (3.9)	<0.001
Waist circumference (cm)	87.3 (10.0)	88.9 (10.4)	91.1 (10.5)	94.3 (10.6)	<0.001
Body fat (%)	27.2 (8.0)	28.8 (8.3)	31.4 (9.0)	29.6 (7.9)	0.004
Glucose (mmol/L)	5.4 (0.5)	5.4 (0.5)	5.5 (0.6)	5.5 (0.6)	0.10
HbA1c (mmol/mol)	35.3 (3.0)	35.6 (3.2)	36.1 (3.5)	35.9 (3.0)	0.15
Total cholesterol (mmol/L)	5.4 (1.0)	5.4 (1.1)	5.6 (1.0)	5.6 (1.1)	0.09
HDL (mmol/L)	1.74 (0.6)	1.70 (0.6)	1.65 (0.6)	1.44 (0.5)	0.002
LDL (mmol/L)	3.2 (0.9)	3.2 (1.0)	3.3 (0.8)	3.5 (0.9)	0.02
Triglycerides (mmol/L)	1.00 (0.5)	1.04 (0.5)	1.25 (0.6)	1.50 (0.8)	<0.001
ALT (U/L)	23.9 (8.7)	25.6 (9.6)	25.9 (13.2)	31.4 (13.4)	<0.001
GGT (U/L)	21.5 (18.2)	20.2 (8.3)	24.2 (26.1)	29.6 (26.7)	0.001
CRP (mg/mL)	1.3 (3.4)	1.6 (2.4)	2.4 (3.8)	3.9 (6.7)	<0.001
Zonulin (ng/mL)	23.2 (2.4)	28.1 (1.1)	32.3 (1.5)	44.5 (21.8)	

BMI: body mass index, ALT: alanine aminotransferase, GGT: gamma-glutamyl transpeptidase, HbA1c: glycated hemoglobin A1c, HDL: high-density lipoprotein, LDL: low-density lipoprotein, CRP: C-reactive protein. Data are represented as mean (SD). * Significance by the Kruskal–Wallis Test.

**Table 6:** The quartile analyses for LBP including all individuals (*n* = 500).

Metabolic Parameter	Q1	Q2	Q3	Q4	*p*-Value *
BMI (kg/m^2^)	24.3 (3.6)	24.2 (2.9)	25.0 (2.9)	25.6 (3.9)	0.004
Waist circumference (cm)	88.3 (10.4)	88.4 (10.5)	92.6 (9.9)	92.4 (11.3)	0.003
Body fat (%)	28.0 (9.1)	28.7 (8.1)	29.2 (7.9)	31.1 (8.4)	0.06
Glucose (mmol/L)	5.4 (0.6)	5.4 (0.5)	5.5 (0.5)	5.5 (0.6)	0.33
HbA1c (mmol/mol)	35.3 (3.2)	35.7 (3.5)	35.7 (3.0)	36.2 (3.0)	0.25
Total cholesterol (mmol/L)	5.3 (1.1)	5.5 (1.0)	5.6 (1.0)	5.5 (1.0)	0.14
HDL (mmol/L)	1.63 (0.6)	1.88 (0.7)	1.54 (0.5)	1.47 (0.6)	<0.001
LDL (mmol/L)	3.2 (0.9)	3.2 (0.9)	3.5 (0.9)	3.4 (0.9)	0.03
Triglycerides (mmol/L)	1.08 (0.6)	1.03 (0.5)	1.26 (0.7)	1.42 (0.8)	<0.001
ALT (U/L)	25.7 (11.0)	24.4 (8.0)	29.1 (12.1)	27.7 (14.5)	0.004
GGT (U/L)	20.3 (13.4)	23.0 (21.1)	26.3 (24.4)	26.1 (24.9)	0.01
CRP (mg/mL)	1.5 (2.7)	1.6 (5.6)	1.9 (2.8)	4.2 (5.5)	<0.001
LBP (µg/mL)	5.7 (2.0)	8.8 (0.5)	10.3 (0.5)	13.7 (2.8)	

BMI: body mass index, ALT: alanine aminotransferase, GGT: gamma-glutamyl transpeptidase, HbA1c: glycated hemoglobin A1c, HDL: high-density lipoprotein, LDL: low-density lipoprotein, CRP: C-reactive protein, LBP: lipopolysaccharide-binding protein. Data are represented as mean (SD). * Significance by the Kruskal–Wallis Test.

**Table 7:** The quartile analyses for sCD14 including all individuals (*n* = 500).

Metabolic Parameter	Q1	Q2	Q3	Q4	*p*-Value *
BMI (kg/m^2^)	24.8 (3.2)	24.7 (3.5)	25.2 (3.5)	24.3 (3.4)	0.31
Waist circumference (cm)	90.3 (10.5)	91.0 (11.2)	91.8 (11.0)	88.6 (9.9)	0.36
Body fat (%)	28.0 (8.1)	27.6 (8.1)	30.9 (8.8)	30.6 (8.3)	0.01
Glucose (mmol/L)	5.4 (0.5)	5.4 (0.6)	5.4 (0.5)	5.5 (0.6)	0.14
HbA1C (mmol/mol)	35.1 (3.2)	35.7 (2.8)	36.0 (2.9)	36.2 (3.7)	0.01
Total cholesterol (mmol/L)	5.2 (1.0)	5.5 (1.0)	5.6 (1.0)	5.7 (1.1)	0.007
HDL (mmol/L)	1.51 (0.6)	1.62 (0.6)	1.68 (0.6)	1.72 (0.6)	0.08
LDL (mmol/L)	3.1 (0.9)	3.3 (1.0)	3.4 (0.9)	3.4 (0.9)	0.05
Triglycerides (mmol/L)	1.27 (0.7)	1.26 (0.8)	1.13 (0.5)	1.14 (0.6)	0.65
ALT (U/L)	27.3 (9.6)	27.9 (12.1)	24.9 (8.7)	26.7 (15.3)	0.43
GGT (U/L)	22.8 (14.8)	27.3 (29.5)	22.9 (22.1)	22.8 (16.9)	0.54
CRP (mg/mL)	1.3 (1.5)	2.0 (3.3)	2.6 (4.1)	3.1 (7.0)	0.04
sCD14 (µg/mL)	1.1 (0.1)	1.3 (0.1)	1.5 (0.1)	1.9 (0.2)	

BMI: body mass index, ALT: alanine aminotransferase, GGT: gamma-glutamyl transpeptidase, HbA1c: glycated hemoglobin A1c, HDL: high-density lipoprotein, LDL: low-density lipoprotein, CRP: C-reactive protein, sCD14: soluble CD14. Data are represented as mean (SD). * Significance by the Kruskal–Wallis Test.

## Data Availability

The data presented in this study are available in the article and supplementary materials.

## Supplementary Materials

The following are available online at https://www.mdpi.com/article/10.3390/metabo11120877/s1: Table S1: Anonymized data set.
